# Complete Genome Sequence of *Aggregatibacter actinomycetemcomitans* Serotype g Strain NUM4039 (JCM 30399)

**DOI:** 10.1128/genomeA.00158-16

**Published:** 2016-03-17

**Authors:** Masanori Saito, Masaaki Hirasawa, Noriko Kuwahara, Tamami Okada, Koji Umezawa, Taira Kobayashi, Masaaki Okamoto, Mariko Naito, Masatomo Hirasawa, Kazuko Takada

**Affiliations:** a Department of Microbiology and Immunology, Nihon University School of Dentistry at Matsudo, Chiba, Japan; b Microbiology and Immunology, Nihon University Graduate School of Dentistry at Matsudo, Chiba, Japan; c Department of Operative Dentistry, Nihon University School of Dentistry at Matsudo, Chiba, Japan; d Department of Special Needs Dentistry, Nihon University School of Dentistry at Matsudo, Chiba, Japan; e Department of Crown Bridge Prosthodontics, Nihon University School of Dentistry at Matsudo, Chiba, Japan; f Department of Translational Research, Tsurumi University, School of Dental Medicine, Tsurumi-ku, Yokohama, Japan; g Department of Molecular Microbiology and Immunology, Nagasaki University Graduate School of Biomedical Science, Nagasaki, Japan

## Abstract

*Aggregatibacter actinomycetemcomitans* is considered to be a major etiological agent of aggressive periodontitis and includes serotype a to g strains. We herein report the first complete genome sequence of *A. actinomycetemcomitans* serotype g strain NUM4039. The genome is 2,382,853 bp in length with a G+C content of 44.34%.

## GENOME ANNOUNCEMENT

*Aggregatibacter actinomycetemcomitans*, a Gram-negative, facultative anaerobic coccobacillus, is an important pathogen related to aggressively progressive periodontal breakdown in adolescents and adults (1). It has been divided into 6 serotypes (a to f) according to the surface carbohydrate antigens (2). The *A. actinomycetemcomitans* NUM4039 (JCM 30399) strain was isolated as new serotype g from the periodontal pockets of patients with chronic periodontitis (3), and its gene cluster was characterized for the synthesis of serotype g-specific antigens (4). We herein describe the genome sequencing of the serotype g strain NUM4039.

Genomic DNA was processed to generate shotgun and 8-kb paired-end libraries, which were sequenced using the 454 GS FLX titanium platform (Roche) provided by Operon Biotechnologies K.K. (Tokyo, Japan). A total of 283,910 reads of 44,022,530 bp, with an average read length of 155 bp and ~18-fold coverage of the genome, were generated.

These reads were assembled into one large scaffold including 44 large contigs (>1,000 bp) with Celera Assembler version 6.1. Gaps were closed through the sequencing of gap-spanning PCR products. Sequence data were analyzed using GENETYX version 11 (Genetyx Co., Ltd., Tokyo, Japan) and *in silico* MolecularCloning version 5.2.66 (In Silico Biology Inc., Yokohama, Japan) genetic information processing software. The sequence was annotated by the DDBJ Microbial Genome Annotation Pipeline (http://migap.ddbj.nig.ac.jp). Coding sequences (CDSs) and RNAs were extracted using MetaGeneAnnotator version 1.0 (5), tRNAscan-SE version 1.23 (6), NCBI BLAST version 2.2.18, and RNAmmer version 1.2 (7).

The complete sequence of the NUM4039 genome consists of a single circular chromosome 2,382,853 bp in length with a G+C content of 44.34%. A total of 2,364 CDSs, 54 tRNAs, and 19 rRNAs in 6 rRNA loci, 2 CRISPRs, and 6 predicted prophage elements were identified in the NUM4039 genome. The 2,299 predicted CDSs were assigned a putative function, while the remaining 65 CDSs have hypothetical ones. No plasmid was identified in NUM4039. The distribution of the predicted genes based on functional categories for the NUM4039 strain was similar to those for the previously sequenced serotype a D7S-1 strain.

### Nucleotide sequence accession number.

The whole-genome sequence of *A. actinomycetemcomitans* strain NUM4039 was deposited in DDBJ/EMBL/GenBank under the accession number AP014520. The version described herein is the first version.
